# Prevalence and risk factors of Hepatitis D virus antibody among asymptomatic carriers of Hepatitis B virus: a community survey

**DOI:** 10.4314/ahs.v22i1.59

**Published:** 2022-03

**Authors:** Uchenna C Okonkwo, Henry C Okpara, Kenneth Inaku, Tony M Aluka, Evaristus S Chukwudike, Yeonun Ogarekpe, Emin J Emin, Osim Hodo, Akaninyene A Otu

**Affiliations:** 1 Gastroenterology/Hepatology Unit, Department of Internal Medicine; 2 Department of Chemical pathology; 3 Department of Family Medicine; 4 Infectious diseases Unit, Department of Internal Medicine, University of Calabar, Calabar, Cross River State, Nigeria

**Keywords:** Hepatitis D virus, Hepatitis B virus, community survey

## Abstract

**Background:**

Hepatitis D virus (HDV) can cause a chronic infection in the presence of hepatitis B surface antigen and contribute to the burden of chronic liver disease especially in regions where chronic hepatitis B virus (HBV) infection is endemic.

**Aim:**

To determine the prevalence and risk factors of HDV among asymptomatic carriers of HBsAg in Cross River State, Nigeria.

**Methods:**

This was a cross-sectional study conducted among apparently healthy adults resident in Cross River State, Nigeria. A structured questionnaire was used to collect socio-demograhic data and risk factors for HBV/HDV infection. Participants blood samples were screened for HBsAg. Samples that were HBsAg positive were further screened for anti-HDVIgM. Statistical analysis was performed using statistical package for social sciences (SPSS) version 20.

**Results:**

A total of 90 HBsAg positive samples were assayed. The prevalence of anti-HDV IgM was 5.6% (95% CI 1.1–10.1). The HDV positive subjects were mostly females (80%), reported family size of >5 members (80%), had female circumcision (75%) and took injections from Non-certified health care practitioners (NCHCPs). None of the assessed risk factors were significantly associated with HDV infection (p >0.05).

**Conclusion:**

Hepatitis D virus is moderately prevalent amongst asymptomatic HBsAg carriers in Cross River State, Nigeria.

## Introduction

Hepatitis B virus (HBV) induced chronic liver disease is unarguably the most important cause of liver related morbidity and mortality in Nigeria and indeed, sub-Saharan Africa.^1^ HBV prevalence is postulated to rise due to missed opportunities for vaccination especially amongst children in rural areas, unwholesome traditional practices, community misconceptions and poor adult HBV vaccination uptake.^2^,^3^,^4^ HBV is endemic in Nigeria with an estimated national prevalence of 12%.^5^ Hepatitis D virus (HDV) also known as the delta virus is an incomplete virus that requires the presence of HBsAg to survive and multiply.

HDV is a circular ribonucleic acid (RNA) virus with a diameter of 36 nm that encodes only one structural protein, the hepatitis delta antigen (HDAg).^6^ HDV infection occurs only in HBsAg-positive individuals, either as acute co-infection leading to clearance of both viruses especially in immune-competent persons or as super-infection in patients with pre-existing chronic hepatitis B (CHB) leading to persistence of the virus.^7^ The later is documented to be more common in Nigeria as the prevalence of HDV is higher in persons with advanced liver disease including hepatocellular carcinoma compared to persons with acute HBV infection or asymptomatic carriers.^8^,^9^ This is most probably due to horizontal transmission in early childhood which is hypothesized to be a more prevalent route ofHBV transmission in Nigeria.^10^ Absence of anti hepatitis B core immunoglobulin M (antiHBcIgM) reliably identifies cases of chronic super-infection.^11^

Although HBV is endemic in Nigeria, data on HDV sero-prevalence are limited. HDV specific antigen (HDV Ag)/antibody (HDVAb) appears in the blood following exposure. The HDVAg appears transiently in about 20% of patients with acute infection. This is followed by anti-HDVIgM and then, anti-HDVIgG. Anti-HDVIgM connotes an acute HDV infection with active HDV replication. It can also be seen in chronic super-infection once there is active replication and can co-exist with HDV IgG^12^,^13^ HDV RNA is a marker of active HDV replication and is present in almost the same frequency in patients with acute co-infection (64%) and super-infection (71%).^14^ The presence of HDV RNA paralleled that of HDV Ag in the first few weeks following infection and tended to disappear by the fourth week in patients with co-infection unlike in those with super-infection where HDV RNA persisted beyond four weeks and resulted in chronic liver disease. HDV super-infection thus, may contribute significantly to the high burden of chronic liver related morbidity and mortality.^14^

The prevalence of HDV infection in Nigeria is not known but is thought to exceed the global prevalence of 5%.15 Studies in South-West Nigeria reported HDVAg prevalence of 6.5 % in patients with CHB associated chronic liver disease and HDVAb prevalence of 4.9% in asymptomatic HBsAg carriers.^16^,^17^ In South-East Nigeria, the prevalence of HDV antibody was reported to be 12.5% in 96 HBsAg positive patients. 8Further analysis of that study showed that patients with acute hepatitis and asymptomatic infection had a lower prevalence of 4.3% while patients with chronic hepatitis, liver cirrhosis and primary liver cell carcinoma had a higher prevalence of 15%. Since HDV is neither routinely screened for in clinical practice nor pre-blood donation in Nigeria, community based screening exercises as undertaken in this study will provide valuable data on the overall contribution of the virus to the burden of liver disease in the region.

## Aim/Objective

We sought to determine the prevalence and risk factors of HDV Ab among asymptomatic carriers of HBsAg in Cross River State, Nigeria.

## Methodology

### Study area

This was a cross-sectional study conducted in nine communities in Cross River State in the Niger Delta region of Nigeria between March 2015 and August, 2016.

### Study design

The study was a cross sectional analytical study

### Study population

The study population comprised healthy males and females aged18 years and above. All the participants provided written informed consent.

### Sample size calculation

The minimum sample size for this study was 162 which was calculated using the formula18; N=Z2 p (1-p)/d2 where Z = confidence interval of 95%, p = prevalence rate of hepatitis B infection taken as 12%5 and d is the desired precision which was 5%. To account for non-response and attrition, 10% of the above figure was added ie 162. + 16 = 178. This was rounded up to 180 persons. The minimum sample size of 180 was recruited from each of the nine randomly selected communities across Cross River State bringing the total sample to 1,620.

### Sampling technique

Cross River State is comprised of three districts namely Southern, Central and Northern senatorial districts. We used a multistage sampling technique to select nine communities drawn from the following local government areas: Calabar south, Akpabuyo, Akamkpa, Abi, Yakurr, Ikom, Obudu, Ogoja and Yala. Advocacy visits were carried out in the various communities prior to recruitment to intimate the residents on the objectives of the study team.

A structured questionnaire was used to collect information on demographic data and risk factors of HBV/HDV viruses from consenting participants. Further details of the recruitment process are provided in prior publication by this study team.^19^ Only those samples that tested positive to HBsAg by an enzyme-linked immunosorbent assay (ELISA) method were included in this analysis.

### Laboratory investigation

The hepatitis B testing was carried out using assay kits manufactured by DRG International Inc.(USA) which were based on horseradish peroxidase (HRP)-conjugated anti-human IgG and multiple recombinant HBsAg antigens. The samples which were positive on the HBsAg testing were further processed for anti-HDVIgM antibodies titre using anti-HDVIgM ELISA produced by Diagnostic Automation, Inc. with catalog no. 1869-12 (Diagnostic Automation, Inc., 21250 Califastreet, Woodland Hills, CA 91367). The anti-HDVIgM ELISA is a solid phase, two-step incubation, antibody capture ELISA method in which polystyrene microwell strips were pre-coated with antibodies directed to human immunoglobulin M proteins. The study participants' serum samples were diluted and added into appropriate microwells. During the first incubation step, the IgM antibodies were captured. After washing out all the components of the sample, the specific anti-HDVIgM captured on the solid phase were detected by the addition of purified HDVAg conjugated to horseradish peroxidase (HRP-Conjugate). During the second incubation step, the conjugated antigens reacted only with the specific HDV IgM antibodies and after washing to remove unbound HRP-conjugate, chromogen solutions were added into the microwells. In presence of the (HDV-IgM)-(HDV antigen-HRP) immune-complex, the colouress chromogens were hydrolyzed by the bound HRP conjugate to blue-colored product. The blue colour turned yellow after stopping the reaction with sulphric acid solution (stop solution). The intensity of yellow colour was measured using a microwell reader set at 450nm. The intensity of the colour measured was proportional to the amount of HDV IgM antibody in the serum sample. Wells containing samples negative for HDV-IgM remained colourless.

### Data analysis

Statistical analysis was performed using the Statistical Package for Social Sciences (SPSS) version 20. Continuous variables were presented as means and standard deviation (SD) while categorical variables were presented as percentages. Chi square was used to test for association between categorical variables and independent sample T-test for association between continuous variables. Statistical significance was established as p<0.05.

### Ethical approval

Ethical approval was obtained from the Cross River State Health Research Ethics Committee (CRS-HREC) with reference number RP/REC/2015/281.

## Results

Of the 120 samples positive for HBsAg, 90 were assayed for anti-HDVIgM because of technical reasons (small volume of samples and limited number of kits). The ages of the HBsAg positive subjects ranged from 20–70years with a mean age of 35±11.9 years. There were 39 males and 51 females. The mean age of males was 35. 87±13.4 years and the mean age of females was34.32 ±10.7years and the difference was not statistically significant (p = 0.55). Only 5 samples were positive for HDV giving a prevalence of 5.6%. There were 4 females and 1 male with mean age of 29.6±7 years. The mean age of HDV negative subjects was 35.4±12 years. The difference between the mean age of HDV positive and negative subjects was not statistically significant (p = 0.152). Majority (80%) of HDV positive subjects were aged 20–39 years. Table 1 shows the demographic characteristics of the HDV positive and negative subjects.

**Table 1:** Demographic characteristics of HDV positive and negative subjects

Variable	HDV positive	HDV negative	Chi sq.	p-val
Age				
20–29	2 (5.4)	35 (94. 6)		
30–39	2 (11.1)	16 (88.9)		
40–49	1 (5.9)	16 (94.1)	1.717	0.78
50–59	0 (0)	10 (100)		
≥ 60	0(0)	3 (100)		
Missing	0(0)	5 (100)		

Gender				
Male	1 (2.6)	38 (97.4)	1.174	0.27
Female	4 (7.8)	47 (92.2)		

Education				
None	0 (0)	2 (100)		
Primary	1 (12.5)	7 (87.5)	0.9	0.82
Secondary	2 (4.7)	41 (96.3)		
Tertiary	2 (5.7)	33 (94.3)		
Missing	0(0)	2 (100)		

Analyses of risk factors for HDV infection showed that 80% of the subjects who were HDV positive were females, had a family size of > 5, and were aged < 40 years. Seventy-five percent (75%) practiced female circumcision and 40% had multiple injections from non certified healthcare practitioners (NCHPs). Only 20% had multiple sexual partners. However, these associations were not statistically significant on univariate analysis (p > 0.05) (Table 2).

**Table 2:** Risk factors associated with HDV positivity

VARIABLE	HDV POSITIVE (%)	HDV NEGATIVE (%)	CHI SQ	P-value
Family size				
<5	1 (20)	24 (28.2)	0.172	0.67
>5	4 (80)	61 (71.8)		
Communal TB				
Yes	1 (20)	16 (18.8)	0.004	0.94
No	4 (80)	69 (81.2)		
Scarification				
Yes	0 (0)	15 (17.6)	1.059	0.3
No	5 (100)	70 (82.4)		
Female circumcision				
Yes	3 (75)	17 (36)	2.557	0.11
No	1 (25)	30 (63.8)		
Injectionfrom NCHPs				
Yes	2 (40)	26 (30.6)	0.195	0.65
No	3 (60)	59 (69.4)		
Sharing of sharps				
Yes	0 (0)	31 (36.5)	2.782	0.09
No	5 (100)	54 (63.5)		
Blood transfusion				
Yes	0 (0)	16 (18.8)	1.145	0.28
No	5 (100)	69 (81.2)		
MSP				
Yes	1 (20)	10 (11.8)	0.299	0.58
No	4 (80)	75 (88.2)		

NHCP- Non-certified healthcare practitioners.

Communal TB- Communal toothbrush

## Discussion

We found that 5.6% of the samples that tested positive for HBSAg were positive for anti-HDVIgM. The global prevalence of HDV antibody is 5% while in sub-Saharan Africa where HBV is hyper-endemic, the prevalence of HDV antibody is 8.39%.^14^ Central and West Africa have the highest burden of HDV in sub-Saharan Africa with prevalence of 25.64% and 7.33% respectively. 20 There is no documentation of the pooled prevalence of HDV in Nigeria but pockets of studies among healthy population and cohorts with liver cirrhosis and hepatocellular carcinoma have documented rates between 0–15%.^8^,^9^,^16^,^17^ Our finding is slightly higher than the 4.9% and 4.3% reported in western and eastern Nigeria respectively.^8^, ^16^ This may be due to variations in HBsAg prevalence in the different regions of Nigeria. The prevalence of HBsAg in Cross River State is 8.8% and is higher than 5.9% and 6.7% prevalence rates reported in communities in eastern and western Nigeria respectively.^19^,^21^,^22^

HDV infection can occur simultaneously with HBV infection because of shared route and risk factors for transmission. Where this occurs in an immune-competent adult, it usually results in sero-clearance of both viruses. However, in children with poorly developed immune systems and in persons with immune suppression, persistence of both viruses is more likely.^23^ Super-infection in an individual with established HBV infection often leads to persistence of HDV virus and aggravation of the underlying liver injury with accelerated progression to liver cirrhosis and hepatocellular carcinoma.^24^ Anti-HDVIgM consistently identifies cases of active HDV co-infection and super-infection unlike HDVAg which appears transiently following acute infection and anti-HDVIgG which is seen only in chronic infection.^13^,^23^ Consequently, screening for HDV IgM is the recommended screening test with follow up HDV RNA testing if screening is positive.^25^

The prevalence of HDV in this study was found to be highest in those aged 20–39 years. A study in India which assessed 450 CHB patients for HDV infection also reported that HBV/HDV co-infection was substantially higher (45.8%) in those aged 21–40 years.^26^ Although, having multiple sexual partners was reported by a minority of the HDV positive subjects, high frequency of HDV infection in the sexually active age group highlights the importance of sexual transmission and the need to educate persons living with CHB on safe sexual practices.^26^ Our study identified other shared risk factors/behaviours for HBV and HDV viruses such as large family size and female circumcision but the association with HDV infection was not statistically significant. This may be due to the small number of HDV positive subjects. The practice of communal use of tooth brush was earlier reported to be significantly associated with HBV infection in Cross River State.^18^ This practice along with female circumcision is more likely to flourish in large-sized families in rural communities and may contribute to horizontal transmission of HBV/HDV infection. Studies in the western world had identified intravenous drug use (IDU) and sexual transmission as major routes of HDV acquisition.^27^

## Conclusion

Hepatitis D virus is moderately prevalent amongst asymptomatic HBsAg carriers screened in several communities in Cross River State. Further research is required to clearly delineate the actual burden of HDV infection in our setting. Screening for HDV antibody is crucial to identify cases of co-infection and super-infection with HBV given the higher risk for developing severe liver injury including cirrhosis and hepatocellular carcinoma among this cohort.

## Limitation

The limitation of the study was that not all HBsAg positive samples were assayed for anti HDVIgM because of limited number of the kits and small quantity of some specimen. Secondly HDV RNA was not done. This would have assisted in identifying those with chronic super-infection with active viral replication.
